# Bibliometric Analysis of Publications in Scopus-Indexed Public, Environmental and Occupational Health Journals Across Scimago Quartiles (2016–2024)

**DOI:** 10.12688/f1000research.174865.2

**Published:** 2026-03-04

**Authors:** Mehran Rostami Varnousfaderani, Shiva Mohammadjani Kumeleh, Neda Izadi

**Affiliations:** 1 Student Research Committee, PhD Student in Epidemiology, Department of Epidemiology, School of Public Health and Safety, Shahid Beheshti University of Medical Sciences, Tehran, Iran; 2 Occupational Health and Safety Engineering, Shahid Beheshti University of Medical Sciences School of Public Health and Safety, Tehran, Tehran Province, Iran; 3 Research Center for Social Determinants of Health, Research Institute for Metabolic and Obesity Disorders, Research Institute for Endocrine Sciences, Shahid Beheshti University of Medical Sciences, Tehran, Tehran Province, Iran

**Keywords:** : Bibliometrics, Scopus, Public health, Occupational health, Environmental health, COVID-19, Scimago quartiles, research trends, Scopus

## Abstract

**Background:**

This bibliometric analysis evaluates health-related research in Public Health, Environmental Health, and Occupational Health (PHEOH) journals indexed in Scopus, categorized by Scimago quartiles (Q1–Q4) from 2016 to 2024. The study aims to identify trends, research productivity, and thematic priorities across these journals.

**Methods:**

From 654 eligible journals, 100 (25 per quartile) were randomly selected. A total of 70,580 documents were retrieved from Scopus and analysed using Microsoft Excel and VOSviewer (v.1.6.20). Co-occurrence analysis of author and indexed keywords was performed separately for each quartile to identify research hotspots, thematic clusters, and trends over time.

**Results:**

Q1 journals contributed the highest proportion of publications (37.7%), followed by Q2 (25.4%), Q4 (22.1%), and Q3 (14.8%). The United States dominated output in Q1–Q3 journals, whereas Pakistan led in Q4. Across all quartiles, “COVID-19” was the most frequent and highly connected author keyword, followed by mental health, SARS-CoV-2, and child-related research. Indexed keyword analysis ranked “humans” highest in every quartile. Topics related to SARS-CoV-2 and mental health received the highest average citations.

**Conclusions:**

The COVID-19 pandemic significantly influenced the research agenda of Public, Environmental, and Occupational Health journals between 2016 and 2024, particularly in higher-quartile outlets. The findings reveal persistent disparities in productivity across journal tiers and geographic regions.

## Introduction

Public, occupational, and environmental health are the driving forces behind this vision, providing the tools to understand health challenges, predict future threats, and design innovative solutions to improve lives. ^1^ The concept of “health” brings together two essential ideas: “Health” representing overall well-being, and “Hygiene” emphasizing prevention and care. At its core, public health is a collective effort to protect and promote the health of populations. From maintaining hygiene in schools and workplaces to ensuring food safety, it addresses a broad spectrum of issues that affect individual and societal well-being. ^2^ Research in this field spans topics such as disease epidemiology, vaccination strategies, health policy-making, and the impact of social factors on health. Advanced techniques like data analysis and epidemiological modeling further empower scientists to tackle global health challenges effectively. ^3^

Occupational health plays a crucial role in ensuring a safer and healthier working environment by predicting, identifying, and managing the risks workers face. It serves as the backbone of the second vital component of the healthcare system. The ultimate goal is to promote both physical and mental well-being for individuals across various professions. ^4^ Within this field, the focus is on critical issues such as assessing and controlling workplace hazards, preventing occupational diseases, and supporting mental health in work settings. ^5^ In high-risk industries such as chemicals, mining, and construction, occupational health is essential for identifying and mitigating these dangers. Furthermore, ergonomics, which emphasizes the design of workspaces to prevent physical injuries and musculoskeletal disorders, is a key aspect that cannot be overlooked in creating healthier work environments. ^6^

Environmental health extensively examines critical factors such as air pollution, water contamination, climate change, and waste management. Managing air pollution presents a significant challenge, as it is directly linked to severe health issues, including respiratory and cardiovascular diseases, as well as cancer. Ensuring access to clean drinking water and effectively treating wastewater are equally vital for preventing infectious diseases and promoting public well-being. ^7, 8^ Furthermore, reducing noise pollution and addressing the health risks associated with urban traffic and environmental noise are essential, particularly in densely populated areas. These risks are known to cause sleep disturbances, increased stress levels, and cardiovascular problems. ^9^ Broadly speaking, research in environmental health lays the groundwork for developing effective public health policies and preventive measures aimed at mitigating the harmful effects of environmental hazards on human well-being.

The importance and complexity of these topics have made reputable journals in Public Health, The significance and intricacy of these topics have established reputable journals in Public Health, Environmental Health, and Occupational Health (PHEOH) essential sources for groundbreaking research. These journals are widely recognized for publishing innovative studies that address critical health issues across various disciplines. PHEOH journals concentrate on high-impact research aimed at improving health outcomes for individuals, workplaces, and communities. Their objective is to connect science with practice, offering solutions to real-world challenges while enhancing our understanding of health and sustainability. ^10^ By emphasizing the interdisciplinary nature of health sciences, these journals promote collaboration among researchers, policymakers, and professionals. They provide a trusted platform for sharing evidence-based insights, helping to shape policies, guide practical applications, and elevate health standards across diverse areas. ^11^

In the dynamic field of research, the ability to track and analyze academic trends plays a crucial role in advancing scientific knowledge. The concept of “bibliometrics,” derived from the Greek terms “Biblio” (book) and “metric” (measurement), provides a framework for quantitatively studying scholarly work. ^12^ As a well-established and valuable research tool, bibliometrics enables the objective evaluation of the growth, distribution, and influence of research across various academic domains. Through the use of advanced statistical and mathematical methods, bibliometrics examines a range of scholarly outputs from journals to articles by identifying emerging patterns, tracking the distribution of research, and mapping the development of specific fields of study. ^13^ Additionally, bibliometric tools allow researchers to gather data from key academic databases such as Scopus, Web of Science, Google Scholar, and PubMed. This data is then organized for analysis, providing clear visual representations that help researchers gain insights into research trends and future directions. ^14, 15^

The position of a journal in quartiles, Q1 to Q4, reflects variability in visibility, citation potential, and editorial standards, among other metrics. It is possible to compare the publication pattern across quartiles to determine whether priorities of research, geographical contributions, and thematic hotspots vary systematically across high-impact and lower-impact journals. Such stratification is of particular relevance in PHEOH, as funding availability, regional health priorities, and global crises can all shape research agendas. Up to now, no study has comprehensively compared the entire Scopus-indexed PHEOH journal landscape across all four quartiles using bibliometric analysis. This gap in prior research limits our understanding of how journal tier might influence topic selection, productivity disparities, and knowledge dissemination in these critical fields. Consequently, the present study had four separate aims:

1. Describe the distribution of publications across Scimago quartiles for PHEOH journals from 2016 – 2024.
2. Identify the most productive countries, institutions, and authors within each quartile.
3. Map research hotspots and thematic clusters via keyword co-occurrence analysis.
4. Compare temporal trends and citation impact for dominant topics across journal tiers.

The overall objective of this work is to provide researchers, editors, and policymakers with data-based insight concerning the changing research landscape of public, environmental, and occupational health based on the two previous objectives.

## Methods

### Journal selection

This study targeted all active journals classified under the Scopus subject category “Public Health, Environmental and Occupational Health” within the broader area “Medicine”. According to the Scimago Journal & Country Rank database (accessed May 2024), 654 journals met these criteria. To ensure balanced representation across impact levels, journals were stratified by their 2023 Scimago Journal Rank (SJR) quartiles (Q1–Q4). From each quartile, 25 journals were randomly selected using a computer-generated random number sequence, resulting in a final sample of 100 journals.

### Rationale for sampling strategy

Although analysing the entire population of 654 journals would have been ideal, resource and time constraints (particularly the manual verification required for a substantial proportion of lower-quartile journals) made full coverage impractical. The stratified random sampling of 25 journals per quartile was chosen to (a) maintain proportional representation across impact tiers, (b) ensure sufficient statistical power for quartile-level comparisons, and (c) remain consistent with similar large-scale bibliometric studies that typically analyse 80–150 journals when examining stratified journal populations. ^16– 18^

### Data retrieval

Scopus was queried between 1–15 June 2024 using the SOURCE-ID of the 100 selected journals. The publication period was restricted to 2016–2024 to avoid incomplete 2025 data. All document types (articles, reviews, editorials, letters, etc.) were included to reflect the complete scholarly output of the journals. A total of 70,580 documents were exported in BibTeX and CSV formats, including title, authors, affiliations, author keywords, indexed keywords, publication year, and citation counts.

### Search strategy transparency

Journal selection was based on the Scopus SOURCE-ID (SRCID) of the randomly selected 25 journals per quartile (Q1–Q4). For each quartile, the following general search syntax structure was applied in Scopus:

(SRCID() OR SRCID() OR … OR SRCID())

AND PUBYEAR > 2015 AND PUBYEAR < 2026

Parentheses were applied to ensure the correct logical grouping of the SOURCE-IDs. The complete list of the 100 selected journals and their corresponding SOURCE-IDs is provided in Supplementary Appendix 1. No restrictions were applied regarding document type, language, or subject subcategory. All document types indexed in Scopus were included to reflect the comprehensive scholarly output of the selected journals. Due to Scopus export limitations, data for each quartile were exported separately in CSV and BibTeX formats and subsequently prepared for analysis.

### Data analysis

Descriptive statistics were performed using Microsoft Excel. Bibliometric mapping was conducted using VOSviewer version 1.6.20. Separate co-occurrence networks were created for author keywords and indexed keywords for each quartile.

The minimum occurrence threshold was iteratively adjusted within a range of 30–50 for each quartile to generate approximately 100 nodes per network, ensuring comparability across quartiles.

Co-occurrence networks were constructed using the full counting method with association strength normalization (default VOSviewer setting). Clustering was performed using the modularity-based algorithm with the LinLog layout to enhance cluster separation and thematic interpretability.

The following visualizations were generated:

• Network visualization with clustering
• Overlay visualization by average publication year
• Overlay visualization by average citations per document

Total link strength (TLS), cluster composition, and temporal trends were extracted for interpretation.

### Data preprocessing

No manual synonym merging or term harmonization was performed before co-occurrence analysis. Author keywords and indexed keywords were analyzed as provided by Scopus to preserve the original author-intended terminology and to avoid introducing subjective bias through manual term aggregation.

Institutional names and author identities were retained as indexed in Scopus without additional normalization. As the primary focus of the study was thematic keyword co-occurrence rather than author-level or institution-level network centrality analysis, raw indexing was considered methodologically appropriate.

### Ethical considerations

Only publicly available bibliometric data were used; no human or animal subjects were involved.

## Results

### Distribution of Journals and Published Documents

Between 2016 and 2024, the 100 selected journals published a total of 70,580 documents indexed in Scopus. The distribution across quartiles was markedly uneven: Q1 journals accounted for 26,583 documents (37.7%), Q2 for 17,869 (25.3%), Q4 for 15,619 (22.1%), and Q3 for 10,509 (14.9%). Thus, the top quartile (Q1) contributed more than one-third of all publications, while Q3 journals had the lowest share.

### Analysis of the Most Productive Countries, Institutes, and Authors

In the Q1 to Q3 categories, the United States was the most productive country, while in the Q4 category, Pakistan was the most productive country.

Analysis of organizations revealed that the European Center for Disease Prevention and Control (ECDC) in Stockholm, Sweden, Harvard Medical School in Boston, United States, Federal Scientific Center for Medical and Preventive Health Risk Management, and the Pak Emirates military hospital National University were the most productive institutes in the Q1 to Q4 journal categories, respectively.

Galea S, with 116 documents, Agarwal A., with 54 documents, Zaitseva N.V., with 51 documents, and Panknin Hardy-TH, with 85 documents, were the most productive authors in the Q1 to Q4 journal categories, respectively.

### Research Hotspots in Public Health, Environmental, and Occupational Health

Co-occurrence analysis was conducted to identify high-frequency keywords that show hot topics in each PHEOH journal category. We performed two separate co-occurrence analyses for each category with respect to the author keywords and index keywords. In all four categories, most author keywords represented the main topics of the documents, while most index keywords indicated the study settings. Based on the analysis of author keywords, we identified 6 (100), 4 (101), 7 (105), and 3 (100) clusters (nodes) for Q1 to Q4 categories, respectively. Co-occurrence analysis using index keywords showed 5 (100), 4 (100), 4 (101), and 6 (101) clusters (nodes) for the Q1 to Q4 categories, respectively.

It is worth noting that for each co-occurrence analysis, various thresholds were set for the minimum number of times a keyword had to appear to achieve approximately 100 nodes on a thematic map. The most common author keyword across all quartile categories (Q1 to Q4) was “COVID-19”. Furthermore, the keyword with the highest Total Link Strength (TLS) represented the primary focus of research within each quartile. In terms of indexed keywords, “humans” had the highest frequency of occurrence across all categories ( Table 1). The top 10 authors and indexed keywords for each quartile, ranked based on frequency, are summarized in Table 1.

**Table 1.** Scimago Categories and Detailed Node Data Derived from Documents Indexed in Scopus.

Journal Category	Nodes (Terms)	Author Keywords				Nodes (Terms)	Index Keywords			
		Weight		Score			Weight		Score	
		Citations	Year	TLS	Occurrences		Citations	Year	TLS	Occurrences
Q1	Covid-19	19.72	2022.01	2078	1287	Human	19.87	2020.33	196146	22567
	HIV	18.02	2019.92	1262	894	Female	21.03	2020.17	127234	10665
	Mental health	20.26	2020.66	897	713	Male	21.55	2020.15	117490	9535
	Public health	18.56	2021.05	1169	632	Article	23.17	2020.47	106891	9510
	Epidemiology	20.24	2020.31	773	532	Adult	21.63	2020.30	109612	8837
	Depression	17.01	2020.65	628	400	Middle-aged	24.33	2019.98	63435	4570
	Harm reduction	15.26	2021.36	383	396	Controlled study	18.80	2020.54	51729	4090
	Surveillance	18.70	2021.34	563	306	Adolescent	23.44	2019.95	53589	3937
	Sars-covid-2	24.03	2021.97	583	262	Major clinical study	22.80	2020.26	55521	3836
	Mortality	15.39	2020.84	301	246	Public health	21.25	2020.09	28447	3695
Q2	Covid-19	11.86	2021.81	904	948	Human	8.50	2020.32	134039	14759
	HIV	6.79	2019.52	1088	771	Article	6.84	2020.79	94040	8897
	Mental health	10.70	2020.98	311	364	Female	8.02	2020.25	91545	7710
	Primary health care	8.81	2020.30	232	353	Male	8.17	2020.22	89863	7515
	Epidemiology	9.39	2020.14	436	347	Adult	7.61	2020.35	84111	6859
	Public health	7.92	2020.42	226	293	Major clinical study	6.96	2020.47	47412	3531
	Prevention	10.05	2020.03	455	271	Cross-sectional study	8.06	2020.45	37288	2958
	Risk factors	8.09	2020.59	230	249	Controlled study	7.29	2020.75	34942	2841
	Children	12	2020.31	200	249	Middle-aged	8.50	2019.67	40611	2895
	Aids	7.95	2017.96	520	223	Brazil	8.23	2019.84	26237	2624
Q3	Covid-19	4.10	2022.14	319	312	Human	6.83	2019.97	56858	7053
	Mental health	6.24	2020.89	200	251	Article	6.61	2020.29	49434	5678
	HIV	6.27	2019.86	126	137	Male	6.70	2019.84	39670	4304
	Evaluation	9.42	2019.64	89	132	Female	6.73	2019.87	36309	3259
	Sanitation	7.36	2020.17	159	126	Adult	6.37	2019.96	35105	3107
	Physical activity	5.79	2020.52	124	118	Controlled study	5.81	2020.37	33664	3017
	Quality improvement	3.14	2020.79	53	115	Middle-aged	7.36	2019.46	14722	1380
	High altitude	9.02	2020.55	73	113	Major clinical study	6.31	2019.97	16983	1317
	Children	3.83	2020.33	99	112	Procedures	9.80	2018.88	15846	1310
	Depression	7.06	2021.34	161	110	Aged	6.56	2019.82	10391	1081
Q4	Covid-19	4.22	2021.94	602	637	Human	5.93	2019.92	48771	5292
	Occupational health	4.62	2021.21	178	185	Article	5.72	2020.33	41597	3939
	obesity	3.51	2020.08	183	179	Male	6.72	2019.99	39166	3890
	epidemiology	3.79	2020.29	151	179	Female	6.67	2020.01	36486	3042
	children	2.15	2020.20	104	176	Adult	6.79	2020.05	36022	3021
	depression	5.80	2021.26	223	168	Middle-aged	9.30	2019.49	34924	2925
	Public health	2.72	2020.35	181	163	Major clinical study	6.88	2020.15	21065	1516
	Mental health	7.64	2021.27	201	158	Controlled study	6.04	2020.47	19684	721
	Risk factors	4.42	2020.27	123	136	Questionnaire	8.29	2020.17	16090	1395
	Quality of life	1.96	2020.44	98	134	Cross-sectional study	7.17	2020.20	12197	985

TLS: Total link strength; Q: Quartile.

To view the analysis based on other keywords, see Figures 1 to 12, and to view the analysis based on the index keyword, see Supplementary File Figures 13 to 24.

**Figure 1.**
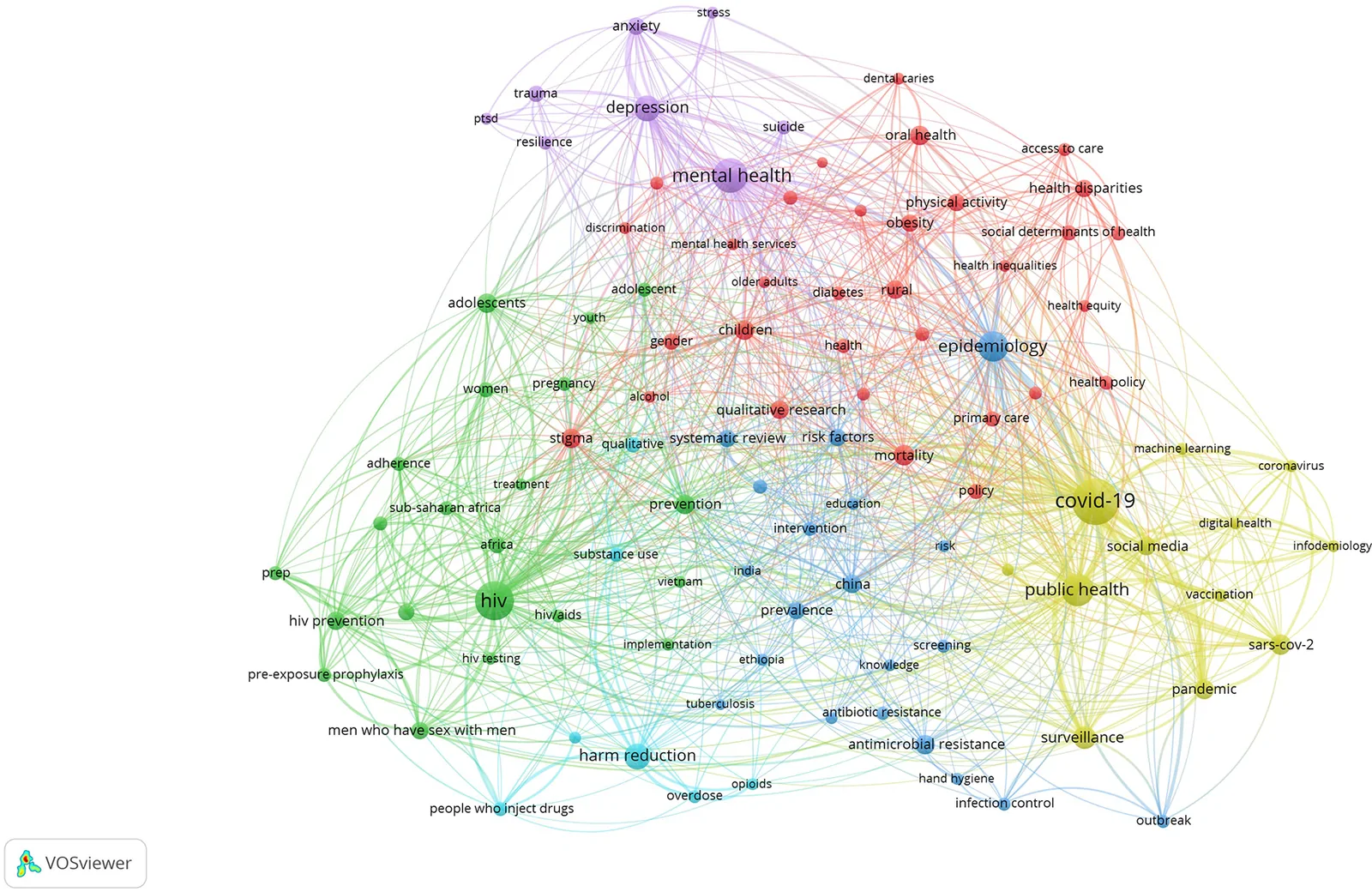
Thematic Map of Document Production in Q1-Category PHEOH Journals: A Co-Occurrence Analysis of Author Keywords.

**Figure 2.**
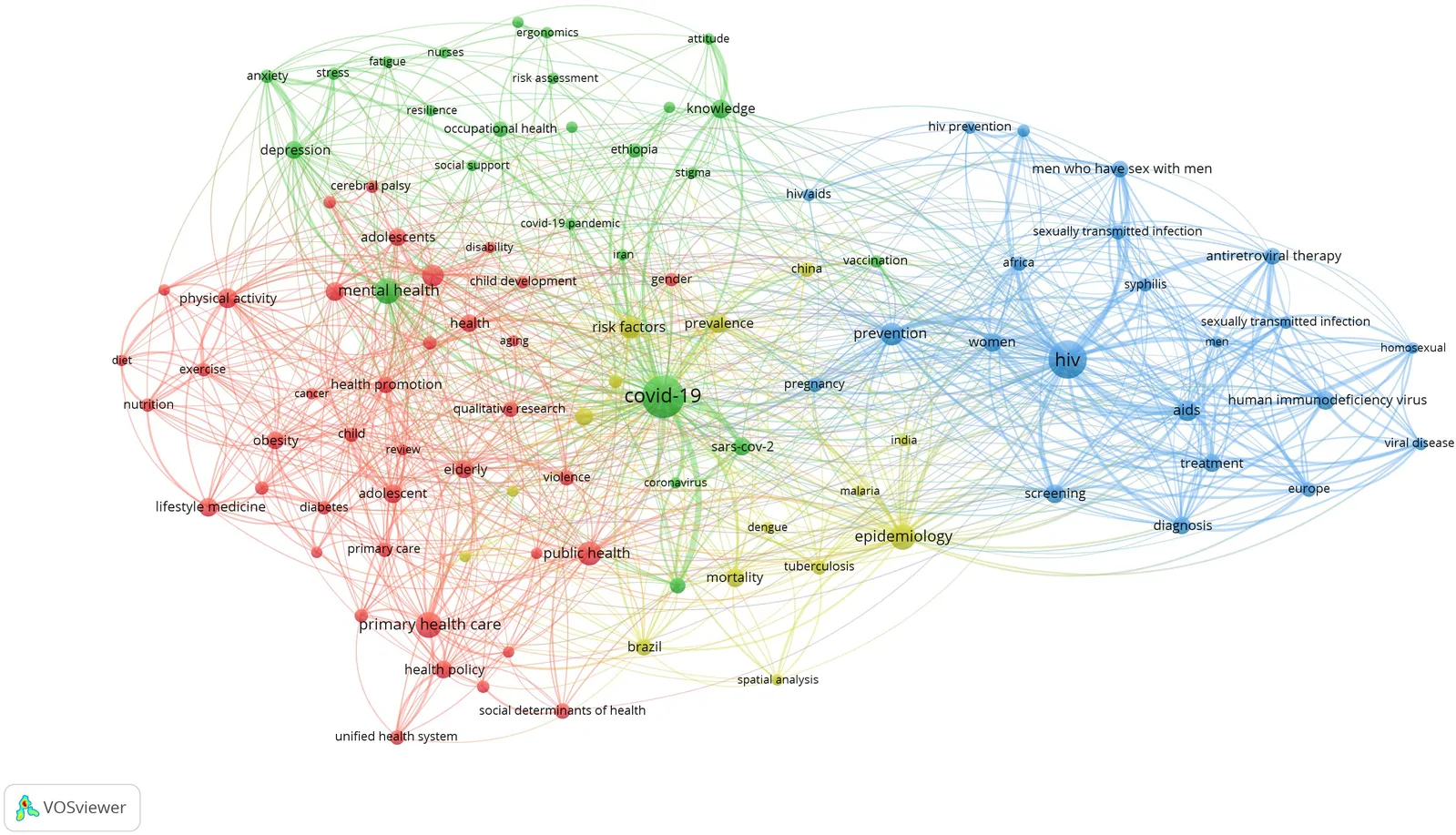
Thematic Map of Document Production in Q2-Category PHEOH Journals: A Co-Occurrence Analysis of Author Keywords.

**Figure 3.**
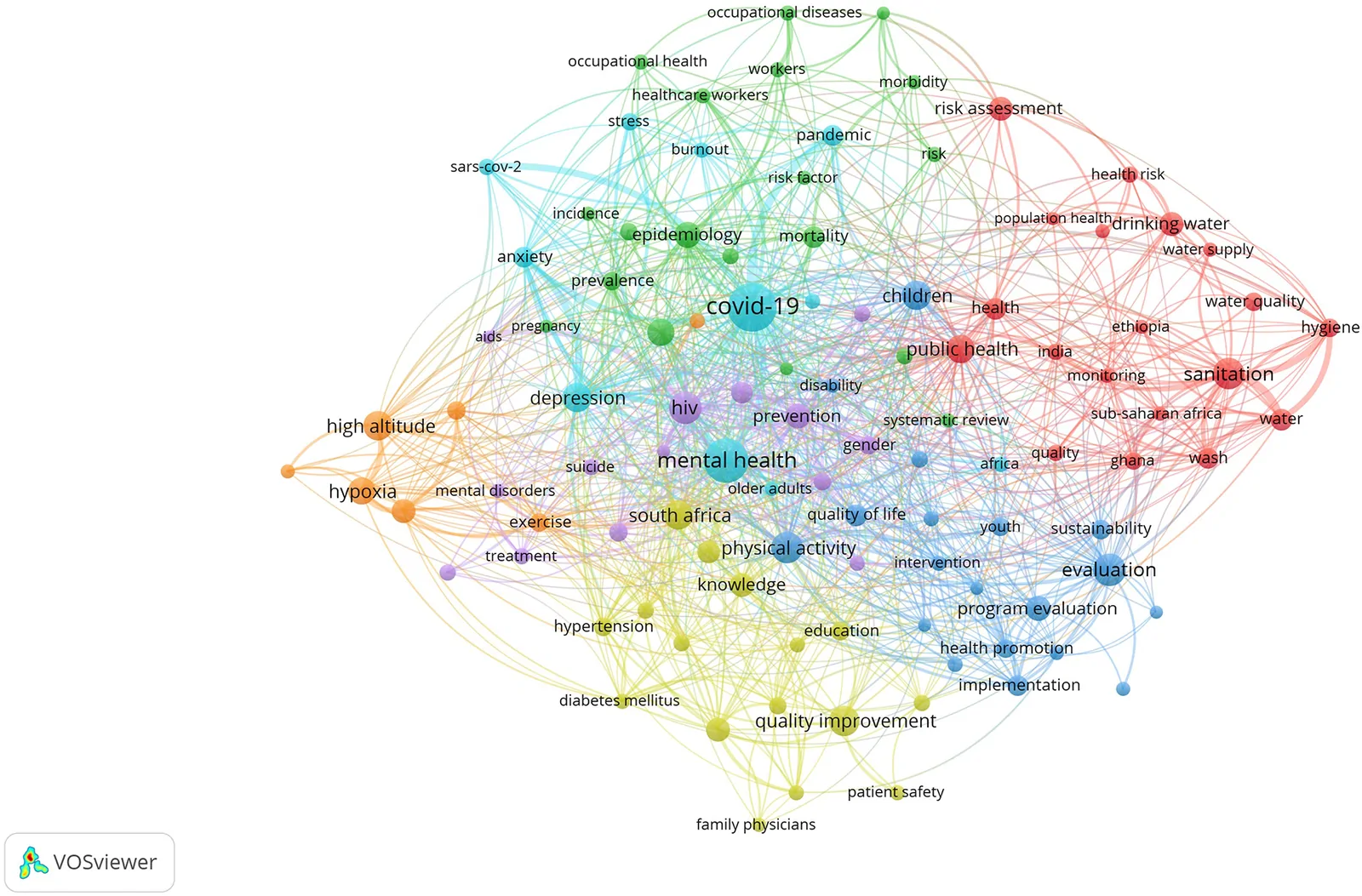
Thematic Map of Document Production in Q3-Category PHEOH Journals: A Co-Occurrence Analysis of Author Keywords.

**Figure 4.**
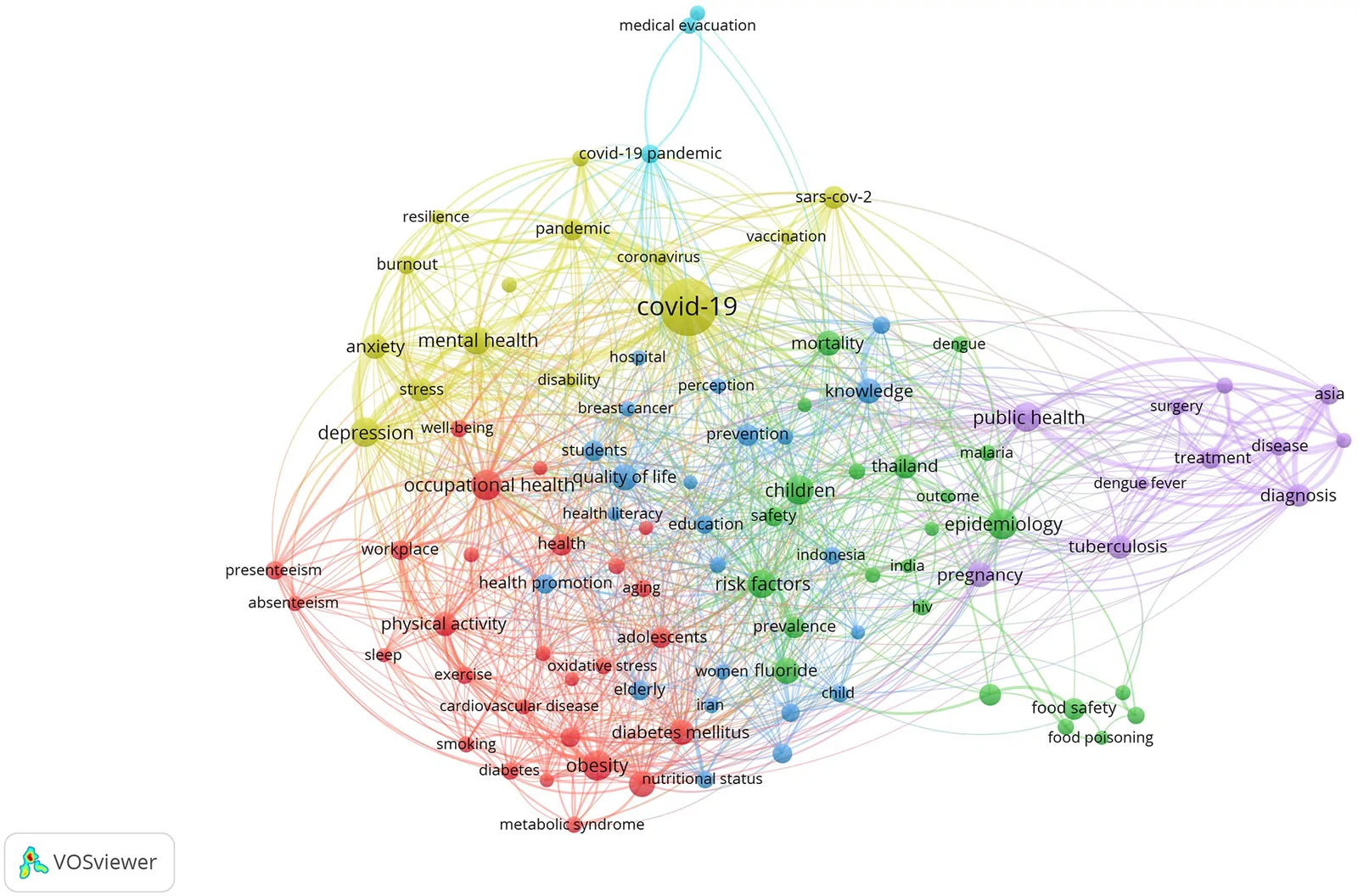
Thematic Map of Document Production in Q4-Category PHEOH Journals: A Co-Occurrence Analysis of Author Keywords.

**Figure 5.**
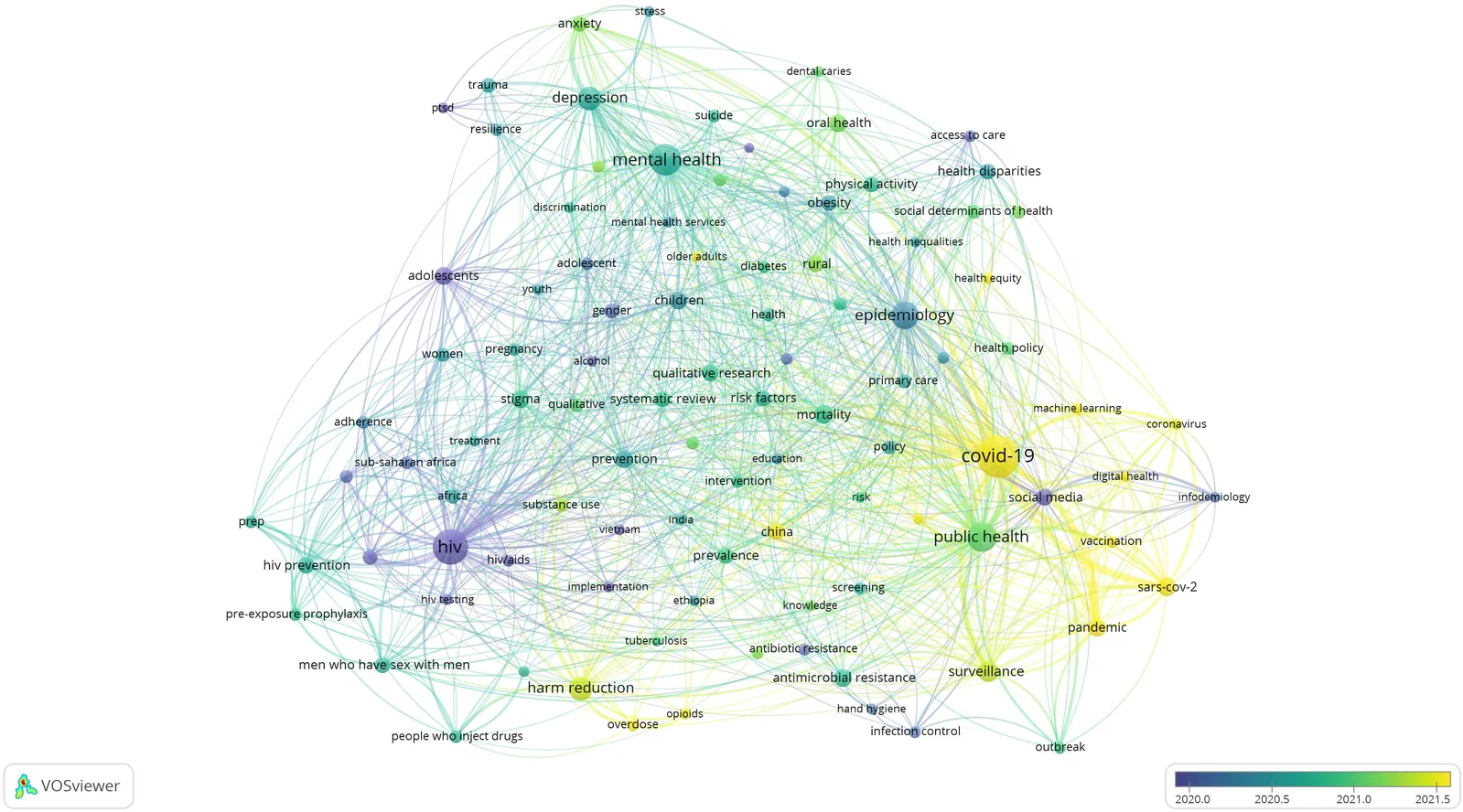
An Overlay Visualization of the Most Frequent Topics in the Q1 Category, Based on Average Publication Year.

**Figure 6.**
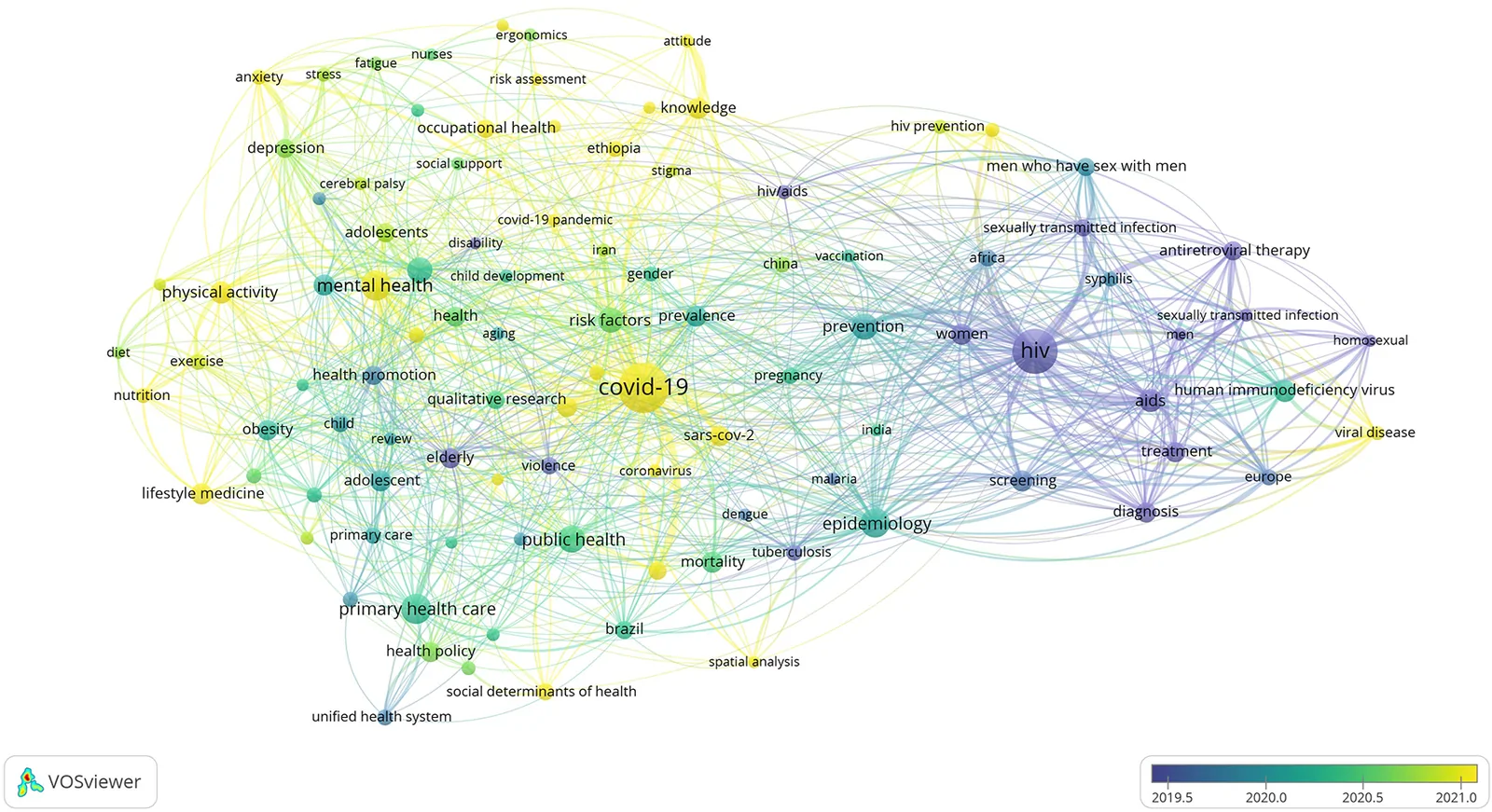
An Overlay Visualization of the Most Frequent Topics in the Q2 Category, Based on Average Publication Year.

**Figure 7.**
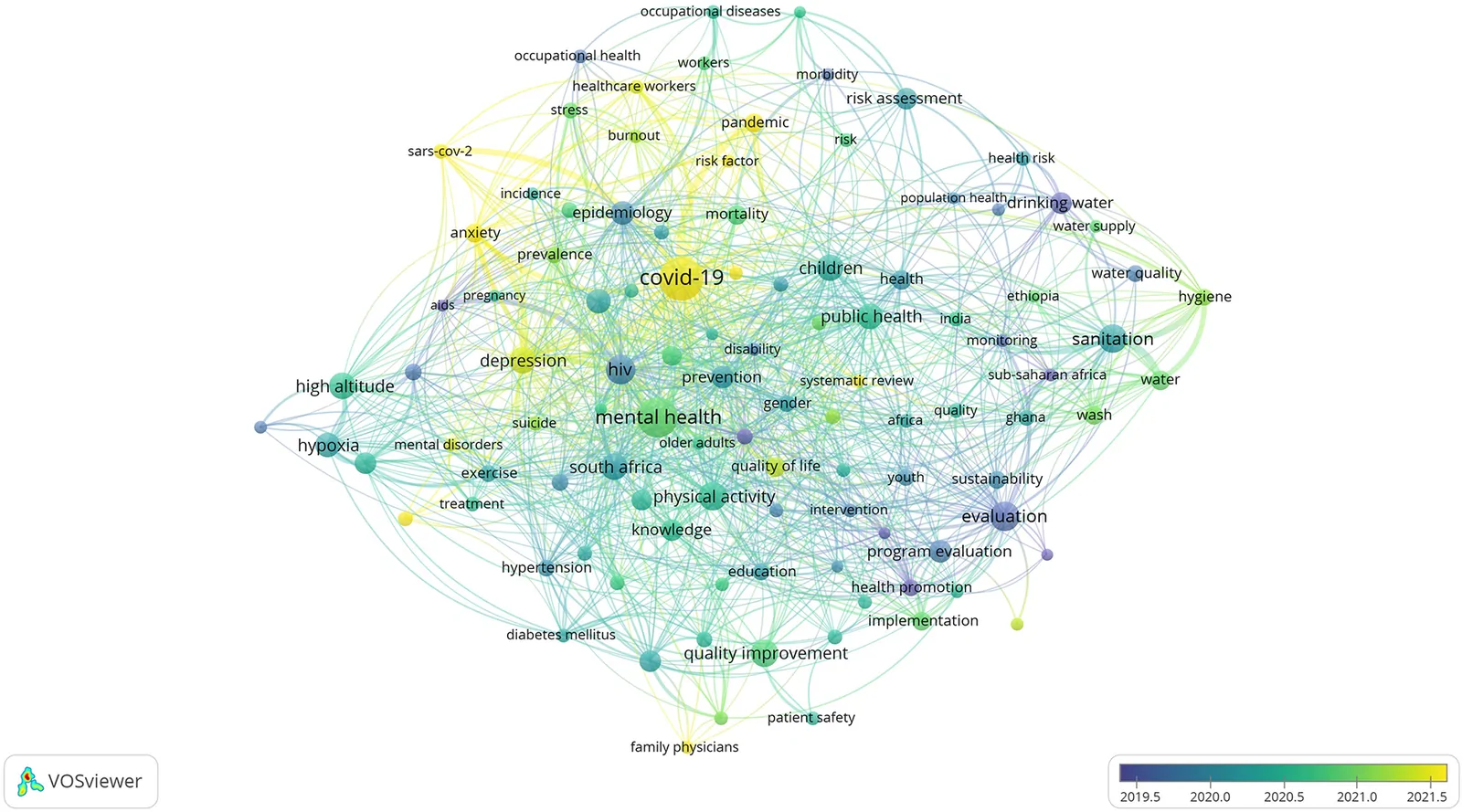
An Overlay Visualization of the Most Frequent Topics in the Q3 Category, Based on Average Publication Year.

**Figure 8.**
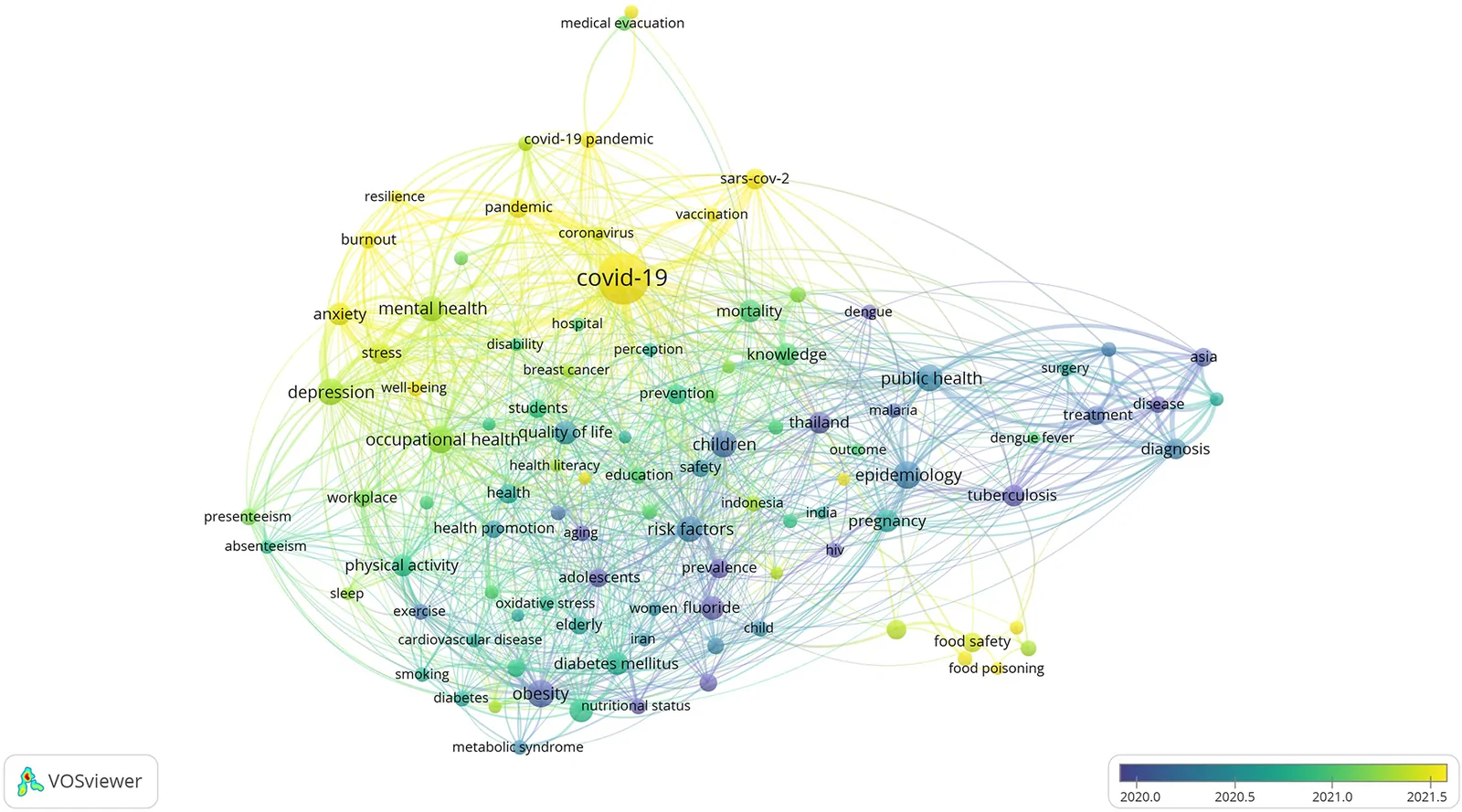
An Overlay Visualization of the Most Frequent Topics in the Q4 Category, Based on Average Publication Year.

**Figure 9.**
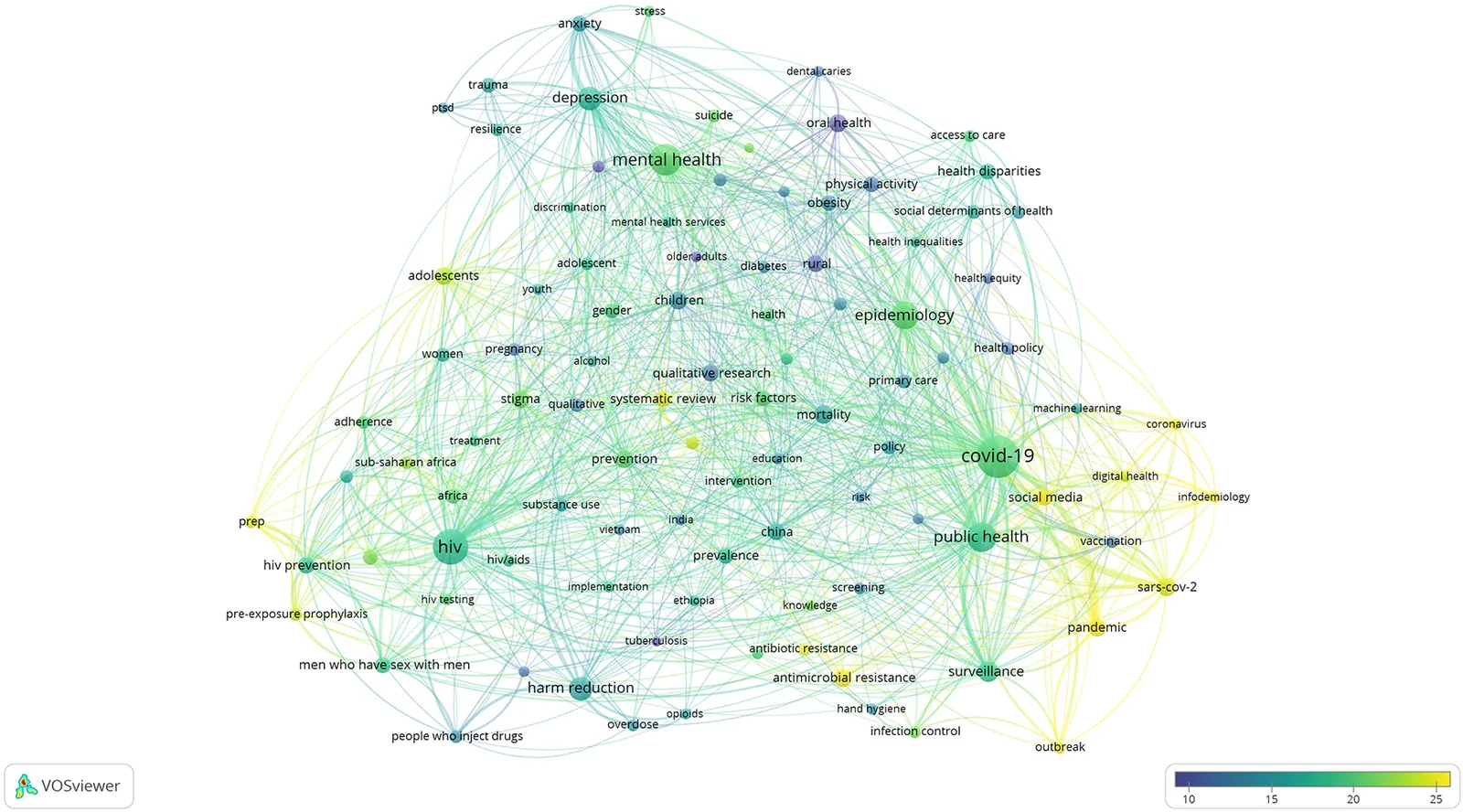
An Overlay Visualization of the Most Frequent Topics in the Q1 Category, Based on Average Citation Counts.

**Figure 10.**
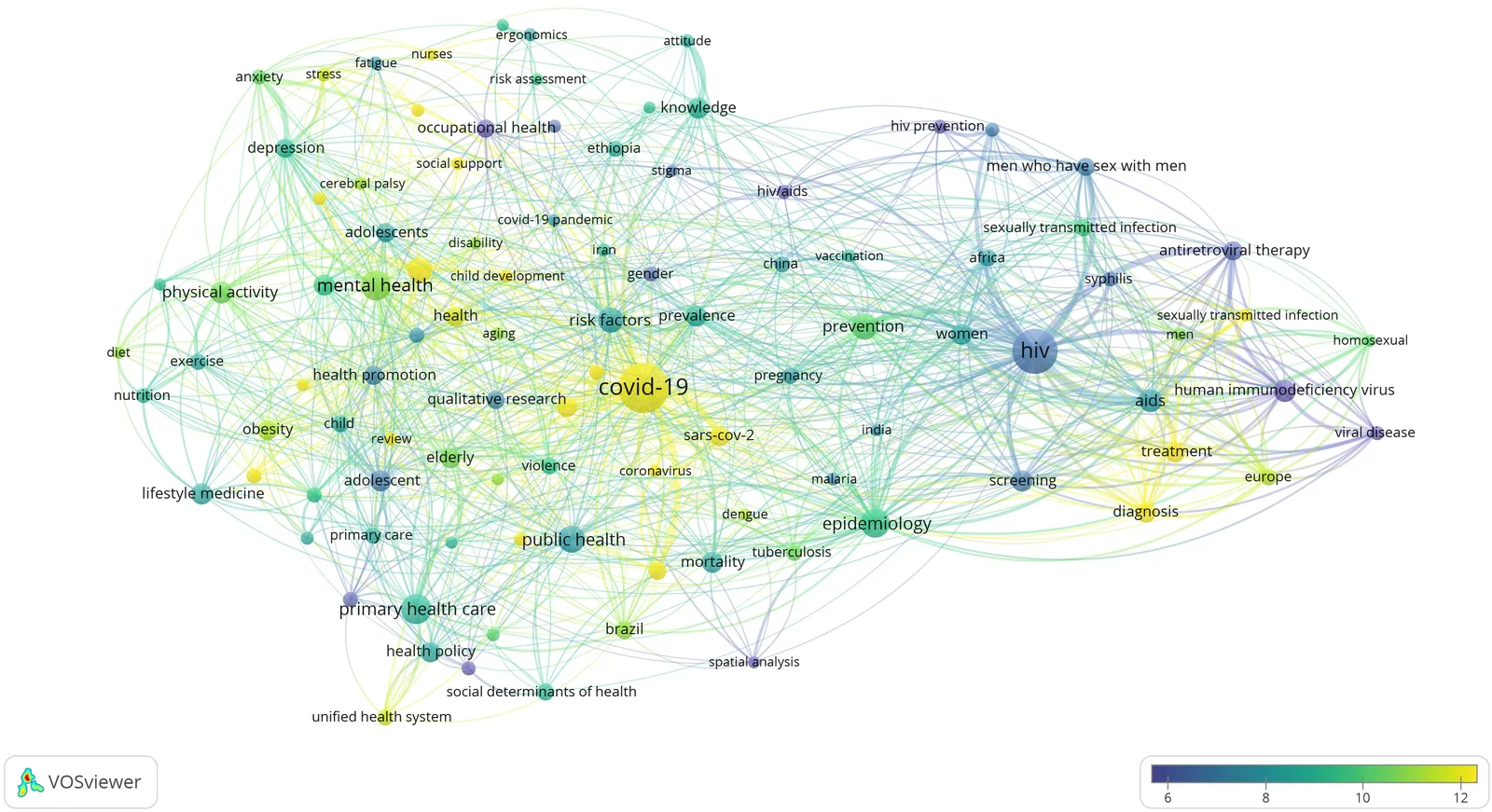
An Overlay Visualization of the Most Frequent Topics in the Q2 Category, Based on Average Citation Counts.

**Figure 11.**
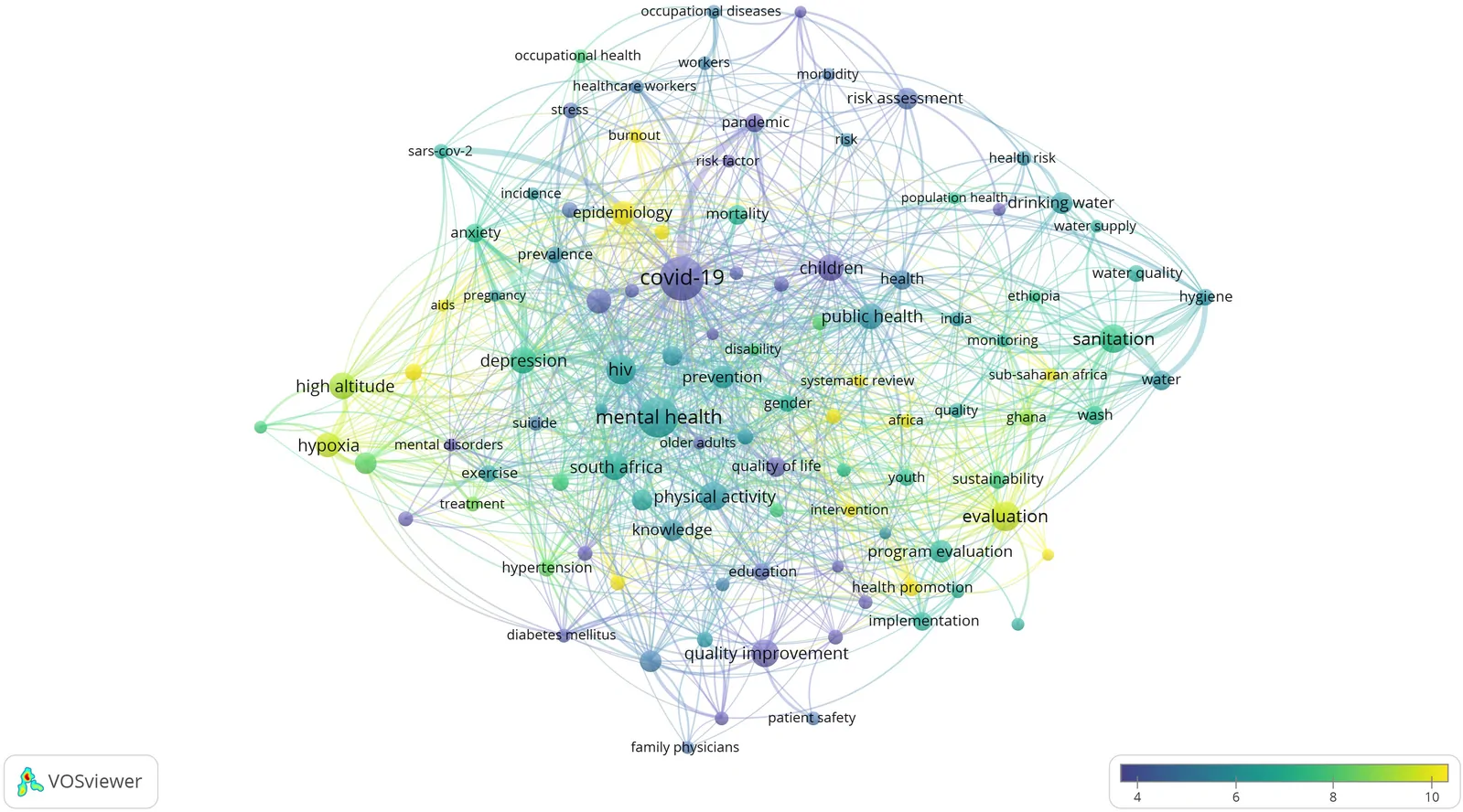
An Overlay Visualization of the Most Frequent Topics in the Q3 Category, Based on Average Citation Counts.

**Figure 12.**
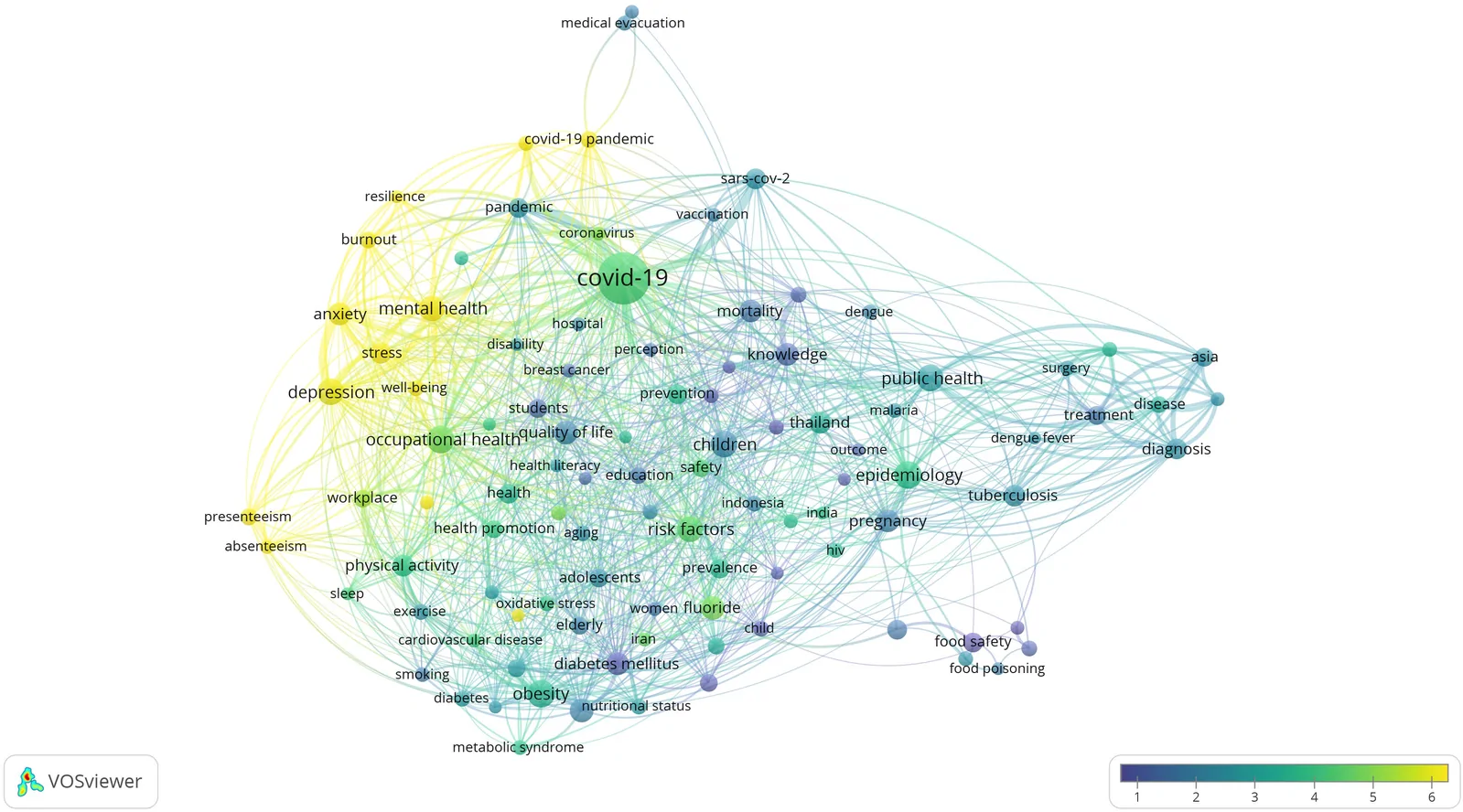
An Overlay Visualization of the Most Frequent Topics in the Q4 Category, Based on Average Citation Counts.

### Temporal trends and citation impact

Among the top 10 author keywords in categories Q1 to Q4, “sars-covid-2”, “children”, “evaluation”, and “mental health” were the most cited with an average of 24.03, 12, 9.42, and 7.64citations, respectively ( Table 1 and Figures 9- 12).

In addition, “COVID-19” was the most up-to-date topic among the top 10 author keywords in categories Q1, Q2, Q3, and Q4 with an average publication year of 2022.01, 2021.81, 2022.14, and 2021.94, respectively ( Table 1 and Figures 5- 8).

## Discussion

This bibliometric study was conducted to explore the bibliometric characteristics of PHEOH journals across different Scimago categories. Our analysis revealed that the most common author keyword in all quartile categories (Q1 to Q4) was “COVID-19”. Additionally, the keyword with the highest Total Link Strength (TLS) represented the central research focus within each category. Notably, “COVID-19” was also identified as the most current topic among the top 10 author keywords in the Q1, Q2, Q3, and Q4 categories. Based on indexed keywords, “humans” had the highest frequency of occurrence across all quartiles. Furthermore, among the top 10 author keywords in Q1 to Q4, terms such as “SARS-CoV-2,” “children,” “assessment,” and “mental health” received the highest citations.

The findings of our study uphold the findings of prior studies: COVID-19 became a focal point in scientific research published from 2016 to 2025. ^19^ Keywords such as “COVID-19,” “SARS-CoV-2,” and “mental health” consistently stood out, which is not surprising given the radical increase in pandemic-related studies during this time. This has also been noted in other bibliometric research works. The preeminence of COVID-19 publications in well-known scholarly works is confirmed by others’ findings, ^19, 20^ who made the same observations about pandemic-related issues in top-tier journals. The common recurrence of “mental health” in Q1 and Q2 journals is probably consistent with widespread concerns about the psychological effects of the pandemic, highlighted. ^21^

Interestingly, our analysis indicated that research productivity was not equal; the USA in Q1 to Q3 and Pakistan in Q4 emerged as the leading contributors. Regional disparities may be associated with differences in funding conditions for research, the quality of research infrastructure, and the respective country’s access to quality journals.

Based on the quartile comparisons and the distribution of publication counts and citation patterns, HIV, mental health, and occupational health emerged as consistently prominent thematic areas within PHEOH journals during the study period. Their high representation and citation impact suggest sustained scholarly attention and thematic centrality within the field.

This prominence likely reflects broader global health priorities and evolving research agendas, particularly in the post-COVID-19 period.

Because our study specifically compares the bibliometric characteristics of Q1 to Q4 PHEOH journals, it stands out as unique. As a result, a direct and comprehensive comparison of our findings with those from previous studies isn’t feasible.

### Limitation

Like any study, this one has its own set of limitations. To begin with, we relied on Scopus Classification Criteria (SjR) for gathering our data. This means we only included documents from journals specifically categorized under public health, environment, and occupation. As a result, any articles published in journals outside these categories or in journals not indexed in Scopus weren’t part of our analysis.

Additionally, because of the sheer number of documents and journals in these categories, combined with a lack of software tools to streamline the process, we had to narrow our analysis.

A total of 654 journals were randomly chosen from a collection of 100 journals covering the period from 2016 to 2025 for our analysis.

Additionally, the study’s methodology restricted our ability to distinguish among the domains of public health, environment, and occupation, which may be regarded as an additional limitation.

## Conclusion

In summary, this bibliometric analysis highlights how research priorities in PHEOH journals differ across Scimago quartiles. The dominant topic within this category is “COVID-19,” with key themes like SARS-CoV-2, “children,” “assessment,” and “mental health” garnering the most citations.

These findings shed light on the current trends and focus areas driving the scientific agendas of PHEOH researchers and journals across various tiers.

## Consent for publication

The authors of this paper have read the final version of the manuscript and approved to submission of the paper to the journal.

## Using Artificial Intelligence Chatbots

None.

## Data Availability

No primary datasets were generated or collected in this study. All analyses were based on bibliographic data retrieved from the Scopus database using predefined journal lists and search strategies. all information required to reproduce the study, including the list of selected journals, the data source, the time frame, and the analytical methods, is fully described in the Methods section. Researchers with legitimate access to Scopus can replicate the analyses by following the procedures outlined in this article.
