# Supplementary material for: Bibliometric Analysis of Publications in Scopus-Indexed Public, Environmental and Occupational Health Journals Across Scimago Quartiles (2016–2024)
Source: F1000Res. 2026 Mar 4;14:1468. Originally published 2025 Dec 27. [Version 2] doi: 10.12688/f1000research.174865.2 (PMC13084236; doi:10.12688/f1000research.174865.2)
Supplement: Peer review history

# Reviewer report: Reviewer response for version 2

**DOI:** 10.5256/f1000research.197007.r468632

**Published:** 2026-04-15

**Authors:** Zulfiya Kannazarova

**Affiliations:** 1 National Research University, Tashkent, Uzbekistan

**General Recommendation: ** Minor Revision (with some moderate methodological clarifications required)

 This manuscript presents a bibliometric analysis of 70,580 documents from 100 Scopus-indexed journals in Public, Environmental, and Occupational Health (PHEOH), stratified across Scimago quartiles (Q1–Q4). Using VOSviewer, the authors analyze publication output, geographic contributions, and keyword co-occurrence patterns. The study highlights the dominance of COVID-19-related research and identifies disparities in productivity across quartiles and countries.

 The topic is relevant and timely, and the manuscript contributes to understanding research trends in PHEOH literature. However, several methodological and interpretative issues require clarification before indexing.

**Major Comments:**

*1. Sampling Strategy and Representativeness*

 The study includes 100 journals (25 per quartile) out of 654 eligible journals. While the authors justify this based on feasibility, concerns remain: The manuscript does not sufficiently demonstrate that the sample is representative of the entire population. Random selection without stratification (e.g., by region, publisher, or subfield) may introduce sampling bias. The equal number of journals per quartile may distort the actual distribution of journals across quartiles.

**Recommendation: **Provide additional justification for sample size and selection method, or include a sensitivity discussion on how results might differ with full dataset inclusion.

*2. Lack of Keyword Harmonization*

 The authors explicitly state that no synonym merging or term standardization was performed. This may lead to fragmentation of conceptually identical terms (e.g., “COVID-19” vs. “SARS-CoV-2”). It potentially affects the accuracy of co-occurrence networks and thematic clustering.

**Recommendation: **Discuss the implications of this choice more critically and consider including a supplementary analysis with harmonized keywords.

*3. Interpretation of Indexed Keywords*

 The finding that “humans” is the most frequent indexed keyword across quartiles is not particularly informative. This likely reflects indexing conventions rather than meaningful thematic trends.

**Recommendation: **Clarify this limitation and avoid over-interpreting such generic indexed terms.

*4. Overemphasis on COVID-19*

 While the prominence of COVID-19 is expected: The manuscript does not sufficiently explore whether this dominance obscures other important long-term research trends. The analysis would benefit from distinguishing pre-pandemic vs. pandemic vs. post-pandemic periods.

**Recommendation: **Include a temporal segmentation analysis or expand discussion on how COVID-19 may have distorted thematic priorities.

Is the work clearly and accurately presented and does it cite the current literature?

Yes

If applicable, is the statistical analysis and its interpretation appropriate?

Yes

Are all the source data underlying the results available to ensure full reproducibility?

Yes

Is the study design appropriate and is the work technically sound?

Yes

Are the conclusions drawn adequately supported by the results?

Yes

Are sufficient details of methods and analysis provided to allow replication by others?

Partly

Reviewer Expertise:

Water ScienceMechanical EngineeringAgricultural Engineering

I confirm that I have read this submission and believe that I have an appropriate level of expertise to confirm that it is of an acceptable scientific standard, however I have significant reservations, as outlined above.

# Reviewer report: Reviewer response for version 2

**DOI:** 10.5256/f1000research.197007.r466978

**Published:** 2026-03-27

**Authors:** Steffen Lemke

**Affiliations:** 1 CAU Kiel University, Kiel, Germany

The authors present a brief descriptive analysis of the state of recent research in Public Health, Environmental Health, and Occupational Health (PHEOH), based on Scopus data and SCImago journal quartiles covering the output of 100 journals over the years 2016-2024. They use VOSviewer to visualize co-occurrences of frequent author- and indexed keywords, particularly focusing on respective works' average publication years and citation counts.

 I believe the article to be methodologically sound and the descriptions of the processes of data collection and processing to be clear. I do however have a number of suggestions that the authors might want to consider for future improvements of the article:

 First, as the SCImago quartiles are of such central meaning to the article's methodology, I believe that adding one or two sentences on what precisely a journal's quartile is based on might help readers to better assess the results. The authors already do hint at journal quartiles typically being about aspects like "visibility, citation potential, and editorial standards", but a bit more concrete information on how the quartiles used in the study are calculated exactly would be helpful.

 Second, the study includes an "Analysis of the Most Productive Countries, Institutes, and Authors", does however provide only a very brief selection of results concerning these aspects, by only mentioning a few outstanding entities from each category. The informative value of the study could be improved substantially by reporting and discussing more comprehensive data from this sub-analysis, e.g., in tabular form.

 Third, the study's discussion remains rather brief. I believe the article could benefit significantly from a more extensive reflection on what the descriptive findings imply about the state of PHEOH research. What can we take from these results for practice? Are there any recommendations for the scholarly community and/or policy makers that the authors can derive from their analysis?

 Also, the article contains a few minor language-related issues that I believe should be addressed to improve its comprehensibility:

1. In the article's very first sentence, what does "this vision" refer to?
2. The structure of the fourth paragraph's first sentence ("The importance and complexity...") seems erroneous.
3. In the Introduction's last sentence, the authors refer to "the two previous objectives" - as the preceding list of aims contains four items, a clarification which two of these are meant here might be helpful.
4. Near the end of the subsection "Research Hotspots in Public Health, Environmental, and Occupational Health", the text at one point mentions "other keywords", where I believe what is meant is "author keywords".

Finally: perhaps that is just me, but I couldn't find the article's supplementary material, which the article refers to in multiple places.

Is the work clearly and accurately presented and does it cite the current literature?

Yes

If applicable, is the statistical analysis and its interpretation appropriate?

Not applicable

Are all the source data underlying the results available to ensure full reproducibility?

Yes

Is the study design appropriate and is the work technically sound?

Yes

Are the conclusions drawn adequately supported by the results?

Yes

Are sufficient details of methods and analysis provided to allow replication by others?

Yes

Reviewer Expertise:

Computer Science; Information Science; Bibliometrics; Scholarly Communcation; Web Science

I confirm that I have read this submission and believe that I have an appropriate level of expertise to confirm that it is of an acceptable scientific standard.

# Reviewer report: Reviewer response for version 1

**DOI:** 10.5256/f1000research.192803.r453053

**Published:** 2026-02-14

**Authors:** Dwi Fitria Al Husaeni

**Affiliations:** 1 Universitas Pendidikan Indonesia, Bandung, West Java, Indonesia

**Article Summary:**

 This article presents a bibliometric analysis of Scopus-indexed journals in the Public, Environmental, and Occupational Health (PHEOH) category based on the Scimago Quartile classification (Q1–Q4) for the period 2016–2024. Of the 654 journals that met the criteria, the authors selected 100 journals (25 per quartile) through stratified random sampling.

 A total of 70,580 documents were analyzed using Microsoft Excel and VOSviewer (v1.6.20). The bibliometric analysis included the distribution of publications across quartiles, the most productive countries, institutions, and authors, keyword co-occurrence analysis (authors and indexed keywords), and temporal trends and citation impact. The results show that Q1 accounts for the largest proportion of publications (37.7%), with the topic "COVID-19" being the most dominant keyword across all quartiles. The keyword "humans" appears most frequently among indexed keywords. Topics related to SARS-CoV-2 and mental health received the highest average citations. The study concludes that the COVID-19 pandemic significantly shaped the PHEOH research agenda across all journal quartiles.

**Evaluation**

1. In general, the article's systematic structure and argumentation flow are quite clear. However, there are weaknesses in the literature's novelty. The proportion of old references is still dominant. Based on the bibliography (pages 14–15), References before 2018 9 articles (43%), References 2018–2021 7 articles (33%), References 2022–2025 5 articles (24%). This means that less than 30% of the references come from the last 3 years (2022–2025). For a bibliometric study discussing trends up to 2024, this proportion is relatively low. The latest literature related to the Development of the latest bibliometric methodology, the structural impact of COVID-19 on global publications, the latest SJR-based journal quartile and stratification analysis, should be increased, especially from 2022–2024. Authors can add a minimum of 5–8 recent (≤3 years) relevant references. Strengthen the Introduction and Discussion sections with a synthesis of the latest literature, not just early COVID-19 studies (2020–2021).
2. The bibliometric design with stratified interquartile sampling is appropriate and logical. The use of VOSviewer for co-occurrence analysis conforms to standard bibliometric practices. However, there are limitations in technical transparency that impact replication.
3. The methods section is not detailed enough to guarantee full replication. Methodological deficiencies include the omission of a list of 100 journals. Furthermore, the authors state that journals were randomly selected from 654 journals. However, they do not provide a list of journal SOURCE-IDs in the article. There is also no appendix listing the selected journals. The search syntax (search string) is not displayed. It is not explained whether filters were applied to specific document types, languages, or affiliation cleaning. Furthermore, the authors do not explain whether the data was exported in a single batch or multiple batches. The keyword threshold determination is also not transparent. The article mentions a threshold of 30–50 to produce ±100 nodes, but does not explain the specific thresholds per quartile. The technical rationale for selecting LinLog/modularity is not explained. It does not explain whether synonym cleaning was performed (e.g., COVID-19 vs. SARS-CoV-2). There is no data preprocessing information. Was institutional name normalization performed? Was author duplication cleaning performed? How were term variations combined (e.g., "covid 19" vs. "covid-19")? The author could add an explanation of the VOSviewer parameters used (counting method, normalization, clustering resolution) when displaying the visualization. Without this information, the study cannot be independently replicated.
4. The conclusions regarding COVID-19 dominance and productivity disparities between quartiles are supported by tables and visualizations (Table 1, Figures 1–12). However, the claim that HIV and mental health topics "have a greater chance of being accepted" (page 10) is speculative and needs to be tempered or supported by additional data. This study makes an interesting contribution by systematically comparing bibliometric characteristics across quartiles, a relatively rare undertaking. The design of the study is relevant, and the methodology is generally sound. However, for the article to be scientifically sound and fully reproducible, significant improvements in the transparency of the methods and updating of the literature are needed.

Is the work clearly and accurately presented and does it cite the current literature?

Partly

If applicable, is the statistical analysis and its interpretation appropriate?

Not applicable

Are all the source data underlying the results available to ensure full reproducibility?

Yes

Is the study design appropriate and is the work technically sound?

Yes

Are the conclusions drawn adequately supported by the results?

Yes

Are sufficient details of methods and analysis provided to allow replication by others?

No

Reviewer Expertise:

bibliometric, Information Systems, Computer Science, Computer Science Education, Learning Technology

I confirm that I have read this submission and believe that I have an appropriate level of expertise to confirm that it is of an acceptable scientific standard, however I have significant reservations, as outlined above.

# Response

**Published:** 2026-02-21

**Authors:** Mehran Rostami Varnousfaderani

**Affiliations:** Epidemiology, Shahid Beheshti University of Medical Sciences School of Public Health and Safety, Tehran, Tehran Province, Iran

1. In general, the article's systematic structure and argumentation flow are quite clear. However, there are weaknesses in the literature's novelty. The proportion of old references is still dominant. Based on the bibliography (pages 14–15), References before 2018 9 articles (43%), References 2018–2021 7 articles (33%), References 2022–2025 5 articles (24%). This means that less than 30% of the references come from the last 3 years (2022–2025). For a bibliometric study discussing trends up to 2024, this proportion is relatively low. The latest literature related to the Development of the latest bibliometric methodology, the structural impact of COVID-19 on global publications, the latest SJR-based journal quartile and stratification analysis, should be increased, especially from 2022–2024. Authors can add a minimum of 5–8 recent (≤3 years) relevant references. Strengthen the Introduction and Discussion sections with a synthesis of the latest literature, not just early COVID-19 studies (2020–2021).

**Answer: We sincerely thank the referee for this valuable suggestion. We agree that including more recent sources would enhance the relevance and scientific credibility of the article. In response, we have carefully updated the sections in question by incorporating more recent studies (2022-2024).**

1. The bibliometric design with stratified interquartile sampling is appropriate and logical. The use of VOSviewer for co-occurrence analysis conforms to standard bibliometric practices. However, there are limitations in technical transparency that impact replication.
2. The methods section is not detailed enough to guarantee full replication. Methodological deficiencies include the omission of a list of 100 journals. Furthermore, the authors state that journals were randomly selected from 654 journals. However, they do not provide a list of journal SOURCE-IDs in the article. There is also no appendix listing the selected journals. The search syntax (search string) is not displayed. It is not explained whether filters were applied to specific document types, languages, or affiliation cleaning. Furthermore, the authors do not explain whether the data was exported in a single batch or multiple batches. The keyword threshold determination is also not transparent. The article mentions a threshold of 30–50 to produce ±100 nodes, but does not explain the specific thresholds per quartile. The technical rationale for selecting LinLog/modularity is not explained. It does not explain whether synonym cleaning was performed (e.g., COVID-19 vs. SARS-CoV-2). There is no data preprocessing information. Was institutional name normalization performed? Was author duplication cleaning performed? How were term variations combined (e.g., "covid 19" vs. "covid-19")? The author could add an explanation of the VOSviewer parameters used (counting method, normalization, clustering resolution) when displaying the visualization. Without this information, the study cannot be independently replicated.

**Answer:** **We sincerely thank the reviewer for highlighting the importance of methodological transparency and reproducibility. We fully agree that bibliometric studies must provide sufficient technical detail to allow independent replication. Accordingly, we have substantially revised and expanded the Methods section to improve transparency. Specifically:**

**Search Strategy Clarification**

**The exact search syntax structure used in Scopus has now been explicitly reported. Parentheses have been specified to ensure the correct logical grouping of SOURCE-IDs. The complete list of the 100 selected journals and their corresponding SOURCE-IDs has been provided in Supplementary Appendix 1.**

**Filtering and Data Export**

**We have clarified that no restrictions were applied regarding document type, language, or subcategory. We have specified that the data were exported separately for each quartile due to Scopus export limitations.**

**Threshold Determination**

**The process of iteratively adjusting minimum keyword occurrence thresholds (range: 30–50) to generate approximately 100 nodes per network has now been clearly described.**

**VOSviewer Parameter Specification**

**The counting method (full counting), normalization method (association strength), clustering approach (modularity-based clustering), and LinLog layout selection have now been explicitly reported.**

**Data Preprocessing Transparency**

**We have clarified that no manual synonym merging or institutional normalization was performed. Keywords were analyzed in their raw indexed form to preserve author-intended terminology and to avoid introducing subjective bias through manual aggregation. This approach enhances reproducibility, as future researchers can replicate the analysis using the same raw database export.**

**These revisions substantially improve the technical transparency of the manuscript and ensure that the study can now be independently replicated. We appreciate the reviewer’s constructive suggestions, which have strengthened the methodological rigor of the manuscript.**

  

1. The conclusions regarding COVID-19 dominance and productivity disparities between quartiles are supported by tables and visualizations (Table 1, Figures 1–12). However, the claim that HIV and mental health topics "have a greater chance of being accepted" (page 10) is speculative and needs to be tempered or supported by additional data. This study makes an interesting contribution by systematically comparing bibliometric characteristics across quartiles, a relatively rare undertaking. The design of the study is relevant, and the methodology is generally sound. However, for the article to be scientifically sound and fully reproducible, significant improvements in the transparency of the methods and updating of the literature are needed.

**Answer: We thank the reviewer for this important observation. We agree that the original wording could imply differential editorial acceptance probability, which was not directly assessed in our study. Accordingly, we have revised the Discussion section to remove speculative language and to clarify that our findings reflect thematic prominence and structural importance within the field rather than acceptance probability.**
